# Epigenetic Regulation of Claudin-1 in the Development of Ovarian Cancer Recurrence and Drug Resistance

**DOI:** 10.3389/fonc.2021.620873

**Published:** 2021-03-22

**Authors:** Zachary R. Visco, Gregory Sfakianos, Carole Grenier, Marie-Helene Boudreau, Sabrina Simpson, Isabel Rodriguez, Regina Whitaker, Derek Y. Yao, Andrew Berchuck, Susan K. Murphy, Zhiqing Huang

**Affiliations:** ^1^ Division of Gynecologic Oncology, Department of Obstetrics and Gynecology, Duke University Medical Center, Durham, NC, United States; ^2^ Division of Reproductive Sciences, Department of Obstetrics and Gynecology, Duke University Medical Center, Durham, NC, United States

**Keywords:** ovarian cancer, DNA methylation, epigenetic, chemosensitivity, tumor xenograft, recurrent ovarian cancer

## Abstract

Over 21,000 women are diagnosed with ovarian cancer (OC) in the United States each year and over half that number succumb to this disease annually, often due to recurrent disease. A deeper understanding of the molecular events associated with recurrent disease is needed to identify potential targets. Using genome-scale DNA methylation and gene expression data for 16 matched primary-recurrent advanced stage serous epithelial OCs, we discovered that Claudin-1 (*CLDN*1), a tight junction protein, shows a stronger correlation between expression and methylation in recurrent versus primary OC at multiple CpG sites (R= –0.47 to −0.64 versus R= -0.32 to −0.57, respectively). An independent dataset showed that this correlation is stronger in tumors from short-term (<3y) survivors than in tumors from long-term (>7y) survivors (R= −0.41 to −0.46 versus R= 0.06 to −0.19, respectively). The presence of this inverse correlation in short-term survivors and recurrent tumors suggests an important role for this relationship and potential predictive value for disease prognosis. *CLDN1* expression increased following pharmacologic inhibition of DNA methyltransferase activity (p< 0.001), thus validating the role of methylation in *CLDN1* gene inhibition. *CLDN1* knockdown enhanced chemosensitivity and suppressed cell proliferation, migration, and wound healing (p< 0.05). Stable *CLDN1* knockdown *in vivo* resulted in reduced xenograft tumor growth but did not reach significance. Our results indicate that the relationship between *CLDN1* methylation and expression plays an important role in OC aggressiveness and recurrence.

## Implication

*CLDN1* gene expression and methylation play an important role in ovarian cancer aggressiveness and this relationship may provide a new therapeutic target.

## Introduction

Epithelial ovarian cancer (EOC) is the fifth leading cause of cancer deaths in women. The American Cancer Society estimates that 21,410 women in the United States will be diagnosed with ovarian cancer (OC) and 13,770 women will die of the disease in the year 2021. The high mortality rate in OC is largely due to late-stage diagnoses as a result of non-specific early symptoms (1). Despite a strong initial response to treatment, most patients will develop recurrent tumors that are often drug resistant (2), resulting in high mortality due to a lack of effective treatments (3). With the development of “omics” technologies, we have gained a deeper understanding of cancer mechanisms, which has led to development of some individualized treatments. However, the mechanisms that drive OC relapse and therapies to effectively delay and ultimately prevent this relapse are still unknown.

Whole genome and transcriptome sequencing technologies have enabled the correlation of individual genomic information with disease risk factors and treatment prognoses. Numerous prior studies have demonstrated that there are gene expression and methylation profiles that correlate with OC aggressiveness and outcome (4), as well as a large number of genes that exhibit altered DNA methylation in this disease (5). However, few studies have utilized matched primary and recurrent OCs. Surgical resection is no longer the standard of care for recurrent OC (6), so the “omics” technologies that hold promise for breakthroughs in the design of individualized treatments are limited due to poor access to recurrent OC tumor samples. The availability of an archived set of primary-recurrent OC paired tumors allowed us to better understand how epigenetic-transcriptomic relationships change as the disease progresses from initial diagnosis to eventual recurrence.

The vast majority of malignant ovarian tumors are epithelial (7). Growing evidence has demonstrated that epithelial-to-mesenchymal transition (EMT) can promote tumor metastasis, invasion, as well as chemotherapy resistance in OC (8). EMT is the process whereby epithelial cells become mesenchymal through loss of their cell–cell adhesion resulting in acquisition of enhanced migration/invasion capabilities (8). Loss of cell-cell adhesion is a fundamental mechanistic component in the progression of primary EOC to metastatic disease and eventual recurrence (9). Tight junctions are multiprotein complexes and they function to regulate cell-cell adhesion in epithelial and endothelial cells (10). Suh et al., showed that *CLDN1* is overexpressed in human hepatocellular carcinoma cells and is capable of promoting the EMT process, suggesting a close relationship between *CLDN1* and EMT (11). There is also evidence that tight junctions are involved in the development of OC spheroids (12). OC cells tend to form spheroids in the peritoneal cavity of advanced OC patients (13). Furthermore, spheroid formation and adhesion to the omentum play significant roles in OC recurrence and chemo-resistance (14). In patients treated for OC, spheroid formation allows cells to resist the effects of chemotherapy, which contributes to eventual recurrence (14).

Tight junction complexes are formed by three gene families: occludins, claudins (*CLDN*s), and junctional adhesion molecules. *CLDNs* contribute both structural and functional factors in these tight junctions. *CLDN1* is expressed in almost all known types of epithelial cells and it plays a major role in the regulation of intercellular permeability (15). Studies have shown the importance of *CLDN* family members in epithelial cell derived cancers, including breast and gastric cancers (16, 17), by examining cancer development, tumor progression, and chemosensitivity. However, tight junctions have not been studied extensively in OC, even though epithelial cells from the fallopian tube have been implicated as the source of high-grade serous carcinoma, the most common and aggressive OC (18). We therefore undertook a deeper investigation of *CLDN*s *in vivo* and *in vitro*, with a particular focus on recurrent and aggressive OC phenotypes.

## Materials and Methods

### Tumor Samples

We used 16 matched primary and recurrent OC tissue sets from patients (mean age at diagnosis, 57.4 years) with stage III/IV serous epithelial OC from the Duke Gynecologic Oncology Tissue Bank. The primary tumor specimens were collected at the time of initial debulking surgery. Recurrent tumor samples were obtained from the same patients during “second-look” surgeries. The time to recurrence ranged from 2 to 65 months (mean, 24 months). Survival ranged from 11 to 105 months (mean, 44 months). Samples were obtained after patients provided written informed consent under protocols approved by the Duke University Institutional Review Board. Patient clinical information is listed in **Table 1**.

**Table 1:** Patient clinical information for matched primary and recurrent ovarian cancers. All tumor pairs showed papillary serous histology on pathology exam.

Tumor ID (Primary/Recurrent)	Final Pathology FIGO Grade	CA125 at End of Primary Therapy	Platinum Sensitivity/Resistance (Primary/Recurrent)	Months to Recurrence Following Surgery	Survival (Months)
**1P/1R**	2	>10	S/S	>6	>36
**2P/R**	1	>10	R/R	>6	>36
**3P/3R**	3	Not detected	S/S	>6	>36
**4P/4R**	3	>10	R/R	>6	≤36
**5P/5R**	2	≤10	R/R	>6	>36
**6P/6R**	3	≤10	R/Unknown (Pt. died of disease)	>6	≤36
**7P/7R**	2	≤10	S/S	>6	>36
**8P/8R**	2	Unknown	R/S	≤6	≤36
**9P/9R**	3	>10	R/R	≤6	≤36
**10P/10R**	2	>10	R/R	>6	≤36
**11P/11R**	3	>10	S/R	>6	≤36
**12P/12R**	2	>10	R/S	>6	>36
**13P/13R**	3	≤10	S/R	>6	>36
**14P/14R**	2	Unknown	Unknown/Unknown	>6	≤36
**15P/15R**	2	Unknown	Unknown/Unknown	>6	≤36
**16P/16R**	2	Not detected	S/S	>6	>36

### The Cancer Genome Atlas (TCGA, https://www.cbioportal.org)

*CLDN1* mutations and expression changes in OC were assessed using a publicly available dataset that included 489 patients with Stage II-IV ovarian serous cystadenocarcinoma with their respective copy number variants, survival data, mRNA expression, and HM27 BeadChip methylation data compared with matched normal variants (19). Data was analyzed using cBioPortal’s online analysis tool. Methylation and expression values were compared using Pearson correlation.

### DNA and RNA Extraction

DNA and RNA were simultaneously extracted from each of the fresh-frozen tissue samples using the AllPrep DNA/RNA Mini Kit according to the manufacturer’s protocol (Qiagen; Germantown, MD; Cat#80204). Nucleic acid concentration and purity were assessed using a NanoDrop™ 2000 spectrophotometer (Thermo Fisher Scientific; Waltham, MA).

### Bisulfite Conversion of DNA

The Zymo EZ DNA Methylation Kit (Irvine, CA; # D5001) was used to perform bisulfite (BS) conversion with 500 ng of genomic DNA according to the manufacturer’s protocol.

### DNA Methylation

The Illumina Infinium HumanMethylation450 BeadChip was used to generate quantitative DNA methylation data using bisulfite modified genomic DNA from the 16 paired primary-recurrent OC specimens by Expression Analysis (Research Triangle Park, NC). This data is publicly available through the Duke Digital Data Repository (20). Existing data (GSE51820) (21) was used for independent validation.

### Gene Expression

Affymetrix Human Genome U133A arrays were used to quantify gene expression by the Duke DNA Microarray Facility using total RNA isolated from 16 paired primary-recurrent frozen ovarian tumor samples. This data is publicly available through the Duke Digital Data Repository (22). An independent Affymetrix U133A gene expression dataset from tumors of women who lived <3 years (n= 26) or >7 years (n= 21) post-diagnosis was used for validation and derived from previously published data (GSE51820) (21). Gene expression for 26 OC cell lines treated for 72 h with 5 µM 5’-aza-2’-deoxycytidine (Decitabine; Sigma-Aldrich; St. Louis, MO; #A3656) was quantified using the Affymetrix HT Human Genome U133A Array and is available from the NCBI GEO web site, accession GSE25428. This data was analyzed using a paired student’s t-test.

### Genome Analysis

The HumanMethylation450 BeadChip data includes 485,512 CpG sites and was analyzed using ChAMP, an integrated 450k analytical platform developed by Morris et al. and maintained by Tian et al. (23, 24). The raw data was normalized based on the default probe normalization method, BMIQ, described by Teschendorff et al. (25). Based on the criteria outline by Morris et al., probes were excluded for having p-values above 0.01 and for having fewer than three bead measurements in at least 5% of samples (24, 26). Probes were excluded for not being true CpG sites and for proximity to known SNPs, as identified by Zhou et al. (27). Additionally, probes were excluded for non-specific hybridization to DNA segments and if they were located on the X chromosome to avoid sex-chromosome methylation patterns (28). Probes were stratified by location relative to CpG islands, including “open sea” regions, “CpG shelves”, “CpG shores”, and “CpG islands as annotated by Illumina. The dataset was analyzed to identify differentially methylated probes between the primary and recurrent data sets, as detailed in the ChAMP pipeline documentation (23). A Bonferroni correction was used with an original alpha level of 0.05 (29). The resulting probe list was annotated with the hg19 genome build using a publicly available R package (30). CpG sites with Illumina-designated gene annotations were retained for analysis.

The 16 paired primary-recurrent samples were also analyzed for RNA expression using the Affymetrix Human Genome U133 Plus 2.0 microarray, which included 22,277 probes. This gene expression data was normalized using the robust multiarray average algorithm (RMA) (31). The Affymetrix gene expression data were analyzed by comparing values for primary and recurrent tumors using a paired student’s t-test (alpha= 0.05), with a Bonferroni correction.

The RMA normalized Affymetrix gene expression data was integrated with the Illumina 450k data based on gene name using Excel. CpG sites from the 450k data and expression data from the RMA normalized gene expression data set were retained for further analysis if the associated gene was present in both data sets. The methylation-expression relationship between the 450k beta values and normalized gene expression was analyzed using Pearson’s correlation. Methylation-expression relationships were quantified independently in the primary and recurrent samples. This produced a set of methylation-expression correlation coefficients and p-values for both primary and recurrent data sets. The list of genes with significant correlation was compared between the primary and the recurrent data sets. The resulting list of genes with differential expression-methylation relationships between the primary and recurrent tumors were analyzed using GATHER, a web-based tool for analyzing gene pathway enrichment (32).

### Cell Culture, Transfection, and Treatment

OC cell lines were chosen for their ability to undergo stable siRNA *CLDN1* knockdown (**Supplementary Figure 1**) unless otherwise indicated. Cells were maintained in RPMI 1640 medium (Sigma-Aldrich; St. Louis, MO) with 10% Fetal Bovine Serum (Thermo Fisher Scientific; Waltham, MA) and 1× Penicillin/Streptomycin (Sigma-Aldrich; St. Louis, MO) at 37°C in a humidified incubator with 5% CO_2_. Cell lines were genetically authenticated with each expansion at the Duke University DNA Analysis Facility to confirm identity of newly prepared freezer stocks. For genetic testing, samples were analyzed at polymorphic short tandem repeat markers using the GenePrint 10 kit (Promega; Madison, WI). Allele sizes were determined using an ABI 3130xl automated capillary DNA sequencer. Cell lines were also tested for mycoplasma at each expansion by the Duke Cell Culture Facility.

Cells were grown to 70% to 80% confluence prior to transfection or treatment. Cells were treated with 300 nm or 900 nm PIKfyve Inhibitor YM201636 (Selleckchem; Houston, TX; #S1219) for 24 h followed by assays for proliferation, migration and spheroid formation. Methodology for treatment of cells with decitabine, a cytosine analog that inhibits DNA methyltransferase (DNMT) activity, was previously published (33, 34). For *CLDN1* knockdown, cells were transfected with HiPerFect non-silencing control siRNA (siCON) (Qiagen #1022076; Valencia, CA) or *CLDN1*-specific siRNAs (si*CLDN*1) (si*CLDN1*-6 and si*CLDN1*-8; Qiagen #SI04136083 and #SI04279114, respectively) according to the manufacturer’s protocol (Qiagen). si*CLDN1*-8 was used for all experiments unless otherwise specified given its superior knockdown efficiency (**Supplementary Figure 2**). For chemosensitivity testing, cells were seeded into 96-well plates and grown to ~70% confluence prior to treatment. Carboplatin and paclitaxel (both from Hospira; Lake Forest, IL) were diluted with 1X phosphate-buffered saline (Sigma-Aldrich; St. Louis, MO) to 10 mg/ml and 6 mg/ml, respectively.

### Proliferation, Migration, and Wound Healing Assays

Following siRNA transfection or YM201636 treatment, cells were analyzed for proliferation with a MTT assay (Promega; Madison, WI), for migration with an invasion/migration assay (Cell BioLabs; San Diego, CA; #CBA-110), and for wound healing using a gap closure assay (Cell BioLabs; San Diego, CA; #CBA-120) per manufacturer protocols. For the wound healing assay, the siCON and si*CLDN*1 cells (SKOV8 and IGROV1) were seeded onto a 24-well plate with specialized inserts that create an even width gap in the cell monolayer when removed (Cell BioLabs; San Diego, CA; #CBA-120). The inserts were gently removed after 24 h when the cells were 90-95% confluent. The cells were washed twice with cold PBS in order to remove any floating cells. Micrographs were taken after the gaps were created. Digital micrographs (40× magnification) were taken at 0, 4, 24, and 48-h after gap generation. The pixel width of the gap was measured three times at six distinct locations across the gap of each well using ImageJ, producing 18 measurements for gap width in each cell line at each time point. The data was normalized using the width of the gap at the initial time point. The measured widths were compared between siCON and si*CLDN*1 at each time point using a paired student’s t-test.

### Stem Cell Selective Culture and Spheroid Formation

Monolayer cells were harvested with TrypLE Express Enzyme (Thermo Fisher Scientific; Waltham, MA; #12604013) 24 h after siRNA transfection when cells were approximately 75% confluent. HEYA8 and DOV13 OC cells were cultured in stem cell selective media consisting of DMEM/F-12 supplemented with 0.4% bovine serum albumin, 10 ng/ml b-FGF, 20 ng/ml EGF, and 5 μg/ml insulin (Sigma-Aldrich, St. Louis, MO) (35–37). Cells were cultured on Corning ultralow attachment plates (Corning, NY) and imaged at 0, 4, and 24-h time points following treatment with DMSO mock control or YM201636 (dissolved in DMSO at 10 mM, SelleckChem; Houston, TX). DMSO was used as a mock treatment control. Micrographs were taken at 40× magnification for spheroid formation using the AxioVision system (Zeiss; Dublin, CA) at 0, 4, and 24 h following culture in stem cell selective media.

### Quantitative Real-Time PCR and Immunoblotting

Quantitative real-time PCR (qRT-PCR) was performed with 100 ng of total RNA isolated from cell lines transfected with si*CLDN*1 using the TaqMan RNA-to-CT One Step Kit (Thermo Fisher Scientific; Waltham, MA; #4392938). Relative expression levels of *CLDN1* (Hs00221623_m1), *CD133* (Hs01009241_m1) and *CD44* (Hs01075862_m1; Thermo Fisher Scientific) were measured using beta-2-microglobulin as an internal loading control (Hs00187842_m1; Thermo Fisher Scientific). qRT-PCR was performed using the 7500 Fast Real-Time PCR System (ThermoFisher Scientific, Grand Island, NY. Cat# 4351106) and data was analyzed using Microsoft Excel (Microsoft Corporation, Redmond, WA).

### Western Blotting

CAOV2, OVCA429, OVCAR5, and SKOV8 OC cells were transfected with siCON or si*CLDN*1 24 h after they were plated in 10 cm petri-dishes and reached 70% confluence. The cells were harvested 72 h post transfection using TrypLE Express Enzyme (Thermo Fisher Scientific; Waltham, MA; #12604013). The whole cell lysate was prepared using RIPA Lysis and Extraction Buffer (Thermo Fisher Scientific; Waltham, MA; #PI89900). Western Blotting was performed using anti-human antibodies against CLDN1 (Sigma-Aldrich; St. Louis, MO, Cat# SAB4503545; rabbit polyclonal), E-cadherin (CDH1), Clone#RM244 (Boster; Pleasanton, CA, Cat# M00063-3; rabbit monoclonal) and Vimentin (Abcam; Cambridge, MA. Cat# ab92547 rabbit monoclonal). GAPDH expression with anti-GAPDH antibody (Santa Cruz Biotechnology, Dallas, TX. Cat# sc-47724; mouse monoclonal) was used as an internal control. Fifty micrograms of cell lysate per sample were used for Western blotting. All antibodies were used at a 1:200 dilution. Western blotting was carried out according to the protocol from Abcam and was imaged using ECL Western Blotting reagents (Promega Corporation; Madison, WI; Cat# W1001) by chemiluminescence.

### Immunocytochemistry

OVCAR5 OC cells were transfected with siCON or si*CLDN*1 and incubated for 72 h. Cells were harvested using TrypLE Express Enzyme (Thermo Fisher Scientific; #12604013) and concentrated onto slides using a cytospin centrifuge. Immunocytochemistry was performed for CLDN1 protein (Sigma-Aldrich; St. Louis, MO, Cat#SAB4503545; rabbit polyclonal) and Vimentin (Abcam; Cambridge, MA. Cat#ab92547; rabbit monoclonal), following previously described protocol (34).

### RT^2^ Profiler PCR Array

One microgram of RNA from siCON or si*CLDN*1-transfected HEYA8 cells was converted to cDNA using SuperScript™ IV First-Strand Synthesis System (ThermoFisher Scientific, Grand Island, NY. Cat# 18091200). The cDNA was used for measuring relative gene expression with the RT² Profiler™ PCR Array for Human Epithelial to Mesenchymal Transition (Qiagen, Germantown, MD; Cat# PAHS-090Z). The array data was generated on a 7500 Fast Real-Time PCR System (ThermoFisher Scientific, Grand Island, NY. Cat# 4351106) and analyzed according to the manufacturer’s instructions (Qiagen, Germantown, MD).

### OC Xenografts

SKOV3 cells were stably transduced with non-target shRNA Control (Sigma-Aldrich; St. Louis, MO. Cat# SHC016V) or *CLDN1*-specific shRNA constructs (Sigma-Aldrich; St. Louis, MO. Cat# HUTR11676) given their ability to produce intraperitoneal (IP) tumors (38). The SKOV3 cells were then transduced with the pGreenFire Lenti-Reporter construct that encodes green fluorescent protein and luciferase under control of the EF1alpha promoter (System Biosciences; Mountain View, CA; Cat# TR010PA/VA-P). For both shRNAs and the pGreenFire Lenti-Reporter, the HEK293 cells and lentivector expression system were used according to methodology previously reported (39). GFP-positive SKOV3 cells were enriched using fluorescence-activated cell sorting (FACS) at the Duke Flow Cytometry Shared Resource. The GFP^+^/sh*CLDN*1 or GFP^+^/shControl (shCON) SKOV3 cells were injected into athymic 6-8-week female nude mice through IP injection (5 × 10^4^/per mouse). Mice were housed five per cage and fed *ad libitum* with standard PicoLab^®^ Rodent Diet 20 (5053 lab diet, LabDiet) by the Duke Laboratory Animal Resource under the supervision of licensed veterinarians. Ten mice each were injected with the non-silencing control (shCTL) or the sh*CLDN*1 GFP^+^ SKOV3 cells. Live imaging was performed weekly on each individual mouse following injection to monitor tumor formation and growth using an IVIS Kinetic system at the Duke Optical Molecular Imaging and Analysis Shared Resource. Each mouse was treated with carboplatin (60 mg/kg IP per mouse, once every 4 days) once its tumor(s) reached a total photon flux signal of 1 × 10^7^ as measured on the IVIS system. Total photon flux was averaged for the group and compared with Mann-Whitney testing. The mouse experiments were repeated using the same parameters. All animal work was approved by the Duke Institutional Animal Care and Use Committee.

## Results

### CLDN1 Expression Is Regulated by DNA Methylation

Tight junctions have been previously implicated in epithelial cell derived cancers, but their role has not been clearly defined in OCs, despite the fact that epithelial fallopian tube cells may lead to high-grade serious OCs (16–18). We sought to characterize the impact of *CLDN1* gene regulation in OCs. While *CLDN1* gene amplification was noted in 14% of samples in the TCGA dataset, no mutations were noted. Further, there was no significant correlation between *CLDN1* methylation and mRNA expression in the primary OC TCGA dataset (Pearson correlation, R= -0.03, p= 0.541).

**Figure 1:**
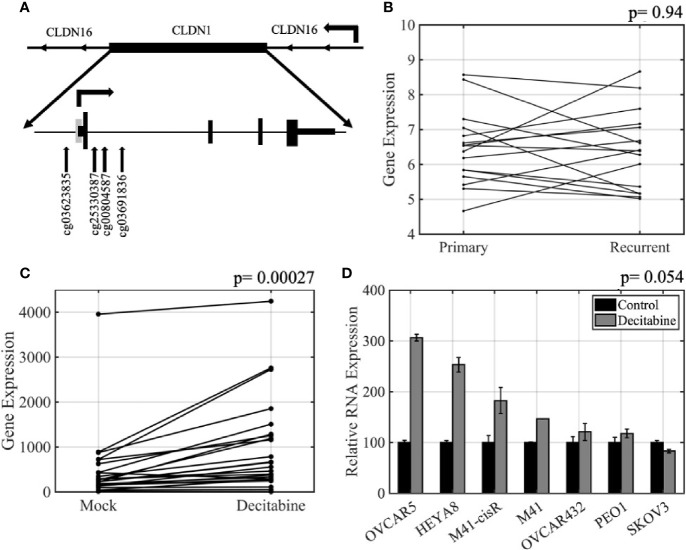
*CLDN* expression is regulated by DNA methylation in ovarian cancer. **(A)** Schematic of the *CLDN1* locus as well as its relative position within a *CLDN16* intron. The positions of the four CpG HumanMethylation Infinium450 BeadChip probes used in this study are shown. Grey rectangle, CpG island; arrow, transcription start; short rectangles, untranslated regions; tall rectangles, coding exons. **(B)** Primary-recurrent robust multiarray average algorithm (RMA) normalized Affymetrix gene expression data for 16 paired primary-recurrent tumors; p = paired t-test. **(C)** *CLDN1* expression in 26 ovarian cancer (OC) cell lines treated with 5 µM decitabine or vehicle for 72 h prior to measuring gene expression; p = paired t-test. **(D)** Quantitative real-time PCR (qRT-PCR) validation showing increased *CLDN1* expression following decitabine treatment; p = paired t-test.

*CLDN1* is located on chromosome 3q28 and has one CpG island which is located at the promoter region. There are 13 methylation probes associated with *CLDN1* on the Illumina Infinium HumanMethylation450 BeadChip assay including six in the promoter CpG island, two in the north shore, one in the south shore, and one in the north shelf (schematic showing relative positions in **Figure 1A**). There were no statistically significant differences for any of the *CLDN1* CpG probes between the matched primary-recurrent OCs, although the methylation levels changed between primary and recurrent OC from each individual patient (paired t-test, p= 0.05–0.99; data not shown). There was no difference in *CLDN1* RNA expression between the primary and recurrent OCs (paired t-test, p= 0.94; **Figure 1B**). There was also no correlation between *CLDN1* RMA expression and patient survival or time to tumor recurrence in 16 primary OC patients (Pearson correlation, R= 0.031, p= 0.89, and R= 0.041, p= 0.86, respectively; data not shown). We then further focused on DNA methylation-expression relationships for *CLDN1* in the matched primary-recurrent OC pairs. The methylation of north shore CpG probe cg00804587 was significantly inversely correlated with *CLDN1* expression in both primary and recurrent OC (paired t-tests, primary OC: R= −0.57, p= 0.02; recurrent OC: R= −0.62, p= 0.009, **Table 2**, **Supplementary Figure 3**). Intriguingly, the methylation status of CpG probes at the north shore (cg25330387), north shelf (cg03601836), and south shore (cg03623835) were most strongly inversely correlated with *CLDN1* expression in recurrent rather than primary OCs (**Table 2**, **Supplementary Figure 3**). Another *CLDN* family member, *CLDN4*, also showed an inverse correlation between expression and methylation at its sole intragenic CpG island in recurrent but not primary OC (cg06350432; Pearson correlation, R= –0.79, p= 0.0002 and R= –0.42, p= 0.11, respectively; data not shown). These data suggest that at least two *CLDN* genes exhibit an enhanced methylation-expression relationship in OC recurrence as compared to primary OCs.

**Table 2:** Claudin-1 (*CLDN1*) methylation-expression relationships in matched primary-recurrent ovarian cancers and short (<3 years) versus long-term (>7 years) survivors.

CpG Probe	Primary OC (n = 16)		Recurrent OC (n = 16)		Short-Term (n = 26)		Long-Term (n = 21)	
	Pearson’s r	p	Pearson’s r	p	Pearson’s r	p	Pearson’s r	p
cg00804587	−0.57	**0.021**	−0.63	**0.009**	−0.19	0.421	−0.41	**0.040**
cg25330387	−0.40	0.123	−0.64	**0.008**	−0.14	0.545	−0.41	**0.037**
cg03601836	−0.32	0.220	−0.62	**0.010**	−0.01	0.969	−0.46	**0.019**
cg03623835	−0.45	0.081	−0.47	0.063	0.06	0.799	−0.41	**0.038**

In the short- and long-term survivor (GSE51820) (21) data analysis, *CLDN1* expression was inversely correlated with DNA methylation in short-term survivors, but not in long-term survivors, at the same probes identified in the matched primary-recurrent data analysis (paired t-test, p= 0.02 to 0.04 versus p= 0.42 to 0.97, respectively; **Table 2**, **Supplementary Figure 3** **)**. All of these CpG probes flank the *CLDN1* promoter CpG island in the 5’ shore (cg03623835), 3’ shore (cg25330387, cg00804587) and 3’ shelf (cg03601836) suggesting that the surrounding regions of the promoter CpG island are potential “hotspots” for tumor control of transcription *via* altering epigenetic regulation. Given that *CLDN1* is located in an intron region of *CLDN16* (**Figure 1A**), we also assessed the methylation-expression correlation for *CLDN16* at 10 CpG sites in both primary and recurrent tumors, but the results were not significant (data not shown). Notably, the TCGA dataset showed an inverse correlation between methylation and expression in CLDN16 for primary OCs (Pearson correlation, R= -0.67, p=1.10e-65).

To further establish the relationship between methylation and expression of *CLDN1*, we analyzed 26 OC cell lines that had been treated with vehicle alone or with 5µM decitabine, a DNMT inhibitor (**Supplementary Table 1**). Affymetrix Human Genome U133A gene expression data showed that *CLDN1* expression increased in the majority of the decitabine treated cells (paired t-test, p= 0.00027; **Figure 1C**). The microarray data was confirmed by qRT-PCR using *CLDN1*-specific primers and probes (**Figure 1D**) with six of the seven tested cell lines showing up to a 3-fold increase in *CLDN1* expression following decitabine treatment (paired t-test; p= 0.0545). Pharmacologic inhibition of DNA methylation did not increase expression of other *CLDN* members (paired t-test, p= 0.08 to 0.91 for *CLDN4*, *CLDN5*, *CLDN6*, *CLDN7*, *CLDN9*, and *CLDN10*; data not shown).

### Chemical and Genetic Inhibition of CLDN1 Suppresses Mobility and Invasion

**Figure 2:**
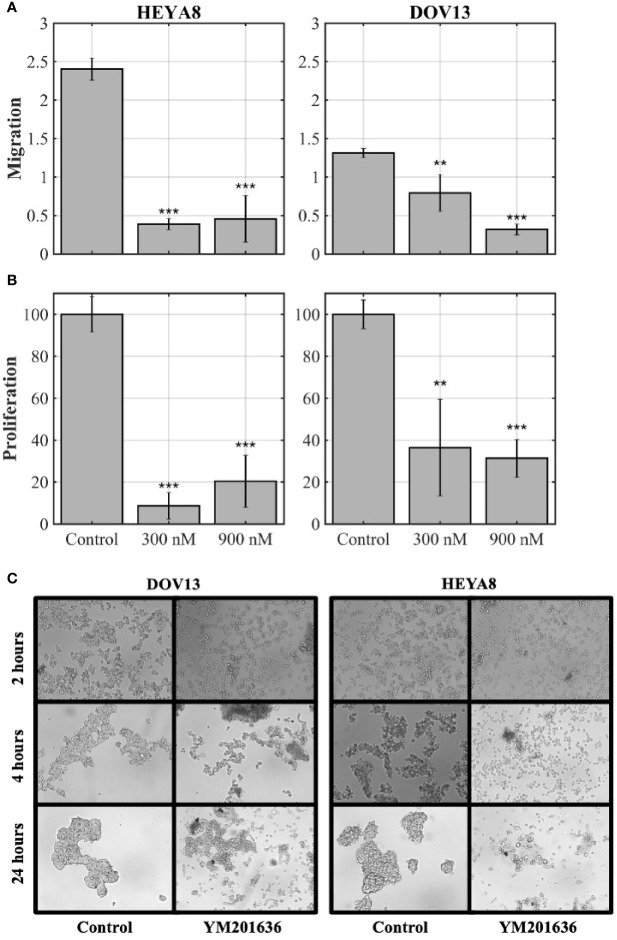
*CLDNs* are functionally involved in cancer cell progression-related behaviors. **(A, B)** Ovarian cancer (OC) cell lines, HEYA8 and DOV13, were treated for 24 h with YM201636 (which blocks the recycling of the tight junction proteins, *CLDN*1/2) followed by migration **(A)** and proliferation (MTT) assays **(B)**; p = two-sample t-tests (*p < 0.05, **p < 0.01, ***p < 0.001). **(C)** OC cell lines (DOV13 and HEYA8) treated with 300 nM YM201636 under stem cell-selective culture conditions with spheroid aggregation assessed at 2-h, 4-h, and 24-h time points after treatment.

Since tight junctions are important for spheroid formation (40), we wanted to determine whether the expression of *CLDN1* was associated with spheroid formation and ovarian tumor progression. Given that HEYA8 and DOV13 cells formed tight spheroids in culture (data not shown), these cell lines were cultured on low attachment plates in stem cell-selective conditions (35). The cells were then treated with 300 nM PIKfyve Inhibitor YM201636, which blocks the continuous recycling of *CLDN1/2* to the cellular membrane and is believed to block their activity in cells (41). Spheroid formation was compared at 2-h, 4-h, and 24-h time points after YM201636 treatment (**Figure 2C**). While tight spheroid formation was observed in mock treated HEYA8 and DOV13 cells, YM201636 treatment disrupted spheroid formation as early as 4 h after treatment as demonstrated by the looser cell aggregates and the presence of single isolated cells and very small aggregates as compared to the controls. Incubating cells with YM201636 for 24 h also led to significant inhibition of cell proliferation (two-sample t-tests, p< 0.01 for all doses and cell lines) and migration (two-sample t-tests, p< 0.01 for all doses and cell lines) as compared to controls (**Figures 2A, B**).

**Figure 3:**
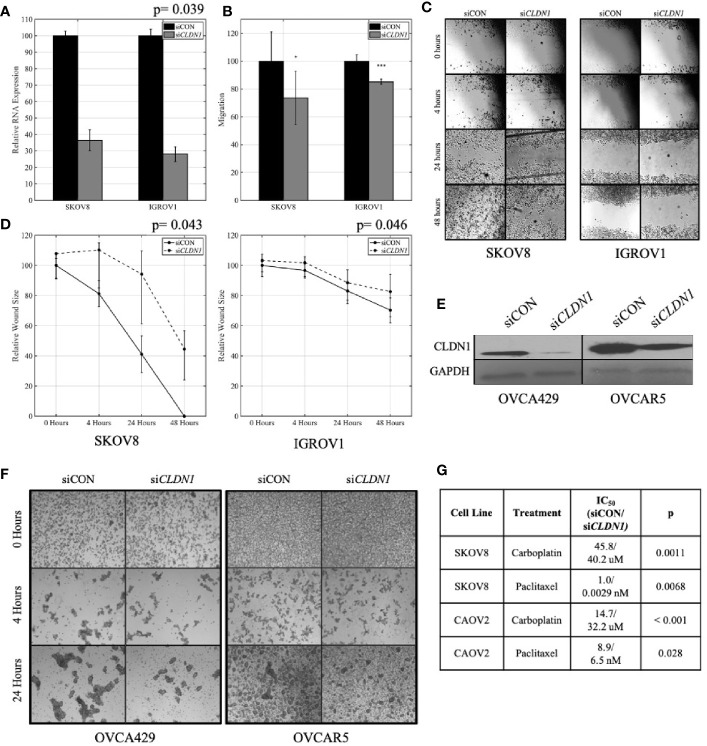
Claudins (CLDNs) are functionally involved in cancer cell progression-related behaviors. **(A)** Knockdown *CLDN*1 expression in ovarian cancer (OC) cell lines, SKOV8 and IGROV1. Real-time PCR (RT-PCR) demonstrated the knockdown efficiency with *CLDN1*-specific siRNAs (si*CLDN*1) versus control siRNA (siCON); p = paired t-test. **(B)** Cell migration assays with *CLDN1* knockdown and control cells; p= two-sample t-test (*p < 0.05, ***p < 0.001). **(C)** Wound healing assay showing a significant decrease in wound healing in si*CLDN1* cells as compared to siCON. **(D)** *CLDN1* knockdown in SKOV8 and IGROV1 OC cells lead to a significant decrease in wound healing capabilities; p = paired t-test. **(E)** Western blot showed significant knockdown of *CLDN1* in OVCA429 and OVCAR5 cell lines used for spheroid formation assay. **(F)** Micrographs taken under 40× magnification to assess spheroid formation at 0, 4, and 24 h following culture in stem cell selective media for siCON and si*CLDN1* treated OVCA429 and OVCAR5 cells. **(G)** Control siRNA (siCON) and *CLDN1*-specific siRNAs (si*CLDN1*) transfected SKOV8 and CAOV2 cells were treated with carboplatin or paclitaxel and IC_50_ values were calculated, showing significant IC_50_ reduction in *CLDN1* knockdown cells; p = paired t-test.

Given that YM201636 treatment is not specific to *CLDN1* alone, we confirmed the role of *CLDN1* by performing similar experiments using siRNA *CLDN1* gene silencing. IGROV1 and SKOV8 both showed significant *CLDN1* knockdown efficiency (**Supplementary Figure 1** **)**, so they were selected to analyze *CLDN1*’s impact on migration and wound healing. The siRNA-mediated knockdown of *CLDN1* expression in 2D culture (paired t-test; p= 0.039; **Figure 3A**) led to slower migration of IGROV1 and SKOV8 (two-sample t-tests, p< 0.001 and p= 0.046, respectively) cells (**Figure 3B**). Wound healing assays for IGROV1 and SKOV8 indicated that *CLDN1* knockdown resulted in delayed gap closure (paired t-test, p= 0.046 and p= 0.043, respectively; **Figures 3C, D**), supporting an important role for *CLDN1* in cancer cell migration. This impact on cell-cell interactions was verified in two additional cell lines, OVCA429 and OVCAR5, which had significant *CLDN1* knockdown efficiency (**Figure 3E**, **Supplementary Figure 1**). Spheroid formation was less efficient following *CLDN1* knockdown in both OVCA429 and OVCAR5 OC cells (**Figure 3F**).

### CLDN1 Knockdown Increases Drug Sensitivity

Since chemoresistance is a very prevalent feature in recurrent OCs, we analyzed chemosensitivity in cells following repression of *CLDN1*. SKOV8 and CAOV2 were chosen given their significant *CLDN1* knockdown efficiency (**Supplementary Figure 1** **)**. siRNA-mediated knockdown led to enhanced sensitivity to carboplatin and paclitaxel treatment in SKOV8 and CAOV2 cells (**Figure 3G**). The IC_50_ values for both drugs were significantly decreased in the SKOV8 *CLDN1* knockdown cells as compared to the same cell lines that received the non-silencing control siRNA (paired t-test, p< 0.01), as well as for paclitaxel in the CAOV2 *CLDN1* knockdown cells (paired t-test, p <0.05; **Figure 3G** **)**. These results were confirmed in SKOV3 for carboplatin using si*CLDN1-6* (IC_50 =_ 362 µM; paired t-test, p= 0.013) and si*CLDN1-8* (IC_50 =_ 404; paired t-test, p= 0.0032) compared to siCON (IC_50 =_ 491 µM), indicating that these results are not due to off-target effects of the siRNA.

### EMT Is Not Involved in CLDN1 Regulation During OC Progression

**Figure 4:**
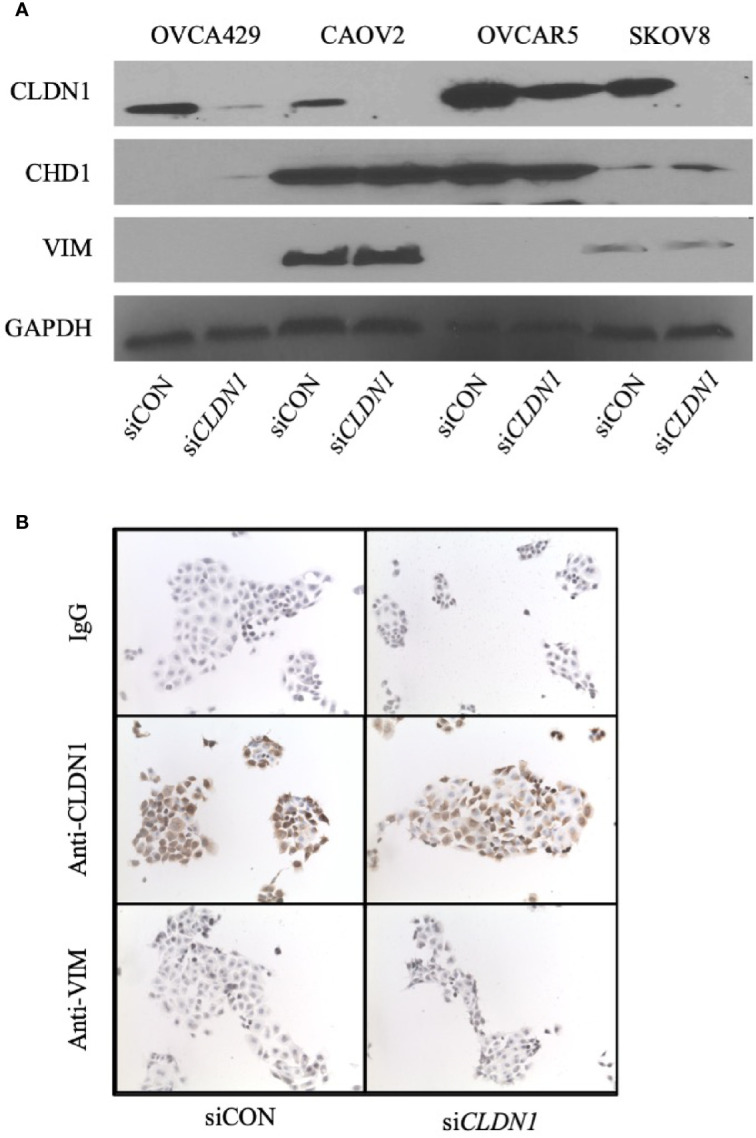
Claudin-1 (*CLDN1*) is not involved in epithelial-to-mesenchymal transition (EMT). **(A)** Four cell lines were transfected with control siRNA (siCON) or *CLDN1*-specific siRNAs (si*CLDN*1) constructs. The cells were harvested 72 h post transfection. Western blotting was performed with antibodies against *CLDN*1, CDH1, Vimentin (VIM) and GAPDH. **(B)** Cytospins of siCON and si*CLDN*1 OVCAR5 knockdown cells were evaluated with antibodies against *CLDN*1 and VIM with IgG used as a negative control.

We sought to determine whether *CLDN1* might be involved in the regulation of EMT in OC, given its role in hepatic cell EMT and potential association with liver metastases (11). The expression of two EMT markers, E-cadherin (CDH1) and Vimentin (VIM), was analyzed using Western blotting and immunocytochemical staining, respectively (**Figure 4**). Western blot for three OC cell lines with *CLDN1* knockdown versus controls showed no change for VIM expression but showed a slight increase in CDH1 expression in SKOV8 cells, a slight decrease in expression in OVCAR5 cells, and no obvious difference in CAOV2 cells (**Figure 4A** **)**. These cell lines were chosen for their demonstrated ability to undergo *CLDN1* knockdown (**Supplementary Figure 1** **).** There were also no differences observed in immunocytochemistry staining for VIM expression in *CLDN*1 knockdown cells versus controls (**Figure 4B**).

To more broadly evaluate potential EMT changes, we used the EMT RT^2^ Profiler PCR Array pre-loaded with primers to evaluate mRNA expression levels for 84 EMT-related genes in HEYA8 with *CLDN1* knockdown (**Supplementary Table 2**. HEYA8 was chosen for this experiment since it had the most substantial *CLDN1* knockdown (**Supplementary Figure 1**). Some EMT genes, including *CAMK2N1*, *GSC*, *SPARC*, and *FGFBP1*, increased their expression >2-fold in *CLDN1* down-regulated HEYA8 cells as compared to controls. However, the primary EMT-associated genes, *CDH1*, *SNAI1*, *BMP1* and *VIM*, showed no significant changes in expression. The lack of primary EMT-associated gene changes in a *CLDN1* knockdown model suggests that *CLDN1* is not a primary mediator in OC EMT progression.

### CLDN1 Knockdown Is Associated With Repression of CD44 and CD133

**Figure 5:**
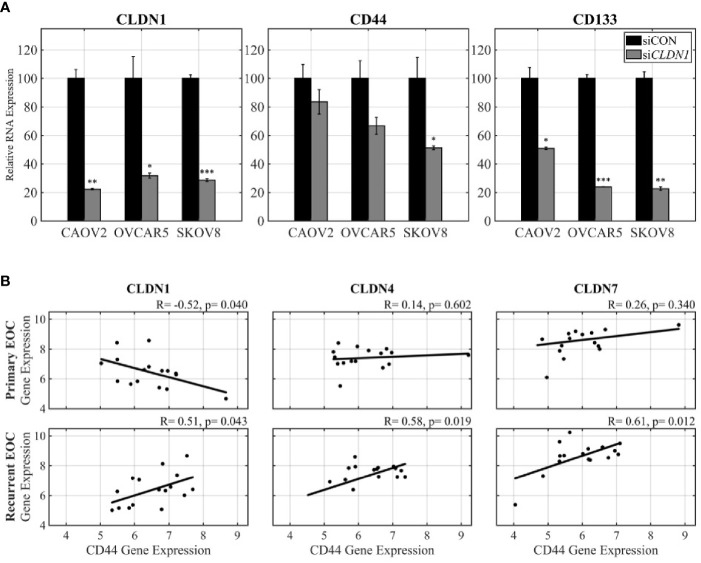
The role of Claudins (*CLDN*s) in OC progression may involve cancer initiating cells. **(A)** Real-time PCR (RT-PCR) was performed for expression of *CLDN1*, *CD44*, and *CD133* in si*CLDN*1 cells in three cell lines; p = two-sample t-test (*p < 0.05, **p < 0.01, ***p < 0.001). **(B)** The microarray dataset from paired primary-recurrent ovarian cancer (OC) was analyzed for co-expression of *CD44* and *CLDN1*, *CD44* and *CLDN4*, and *CD44* and *CLDN7* [x-axis, robust multiarray average algorithm (RMA) normalized expression values of CD44; y-axis, RMA normalized expression values of *CLDN*s]. Pearson correlation and p-values are shown.

We have shown that *CLDN1* is important for spheroid formation using *CLDN*1/2 trafficking inhibition by YM201636 (**Figure 2C** **)** and *CLDN1* knockdown (**Figure 3F**). Increased expression of Cluster of Differentiation 44 (CD44) occurs in OC during the development of metastasis, recurrence and drug resistance (42). Spheroid formation is an important feature of cancer-initiating cells (CIC) and a number of cell surface molecules have been associated with ovarian CICs, including CD44 and CD133 (43). We therefore investigated associations between *CLDN*1 and these CIC cell markers. CAOV2, OVCAR5, and SKOV8 cells were chosen for their significant response to *CLDN1* knockdown (**Supplementary Figure 1**). Real-time RT-PCR of *CD44* and *CD133* expression in CAOV2, OVCAR5, and SKOV8 cells with *CLDN1* knockdown versus control cells showed *CD44* and *CD133* transcription products were both repressed (**Figure 5A**). These findings suggest a potential relationship between *CLDN1* expression and the expression of these OC CIC markers.

The Affymetrix microarray data from the 16 matched primary-recurrent OC tissues showed an inverse correlation between *CD44* and *CLDN1* expression in primary tumors (Pearson correlation, R= −0.52, p= 0.04) but this relationship surprisingly changed to a positive correlation in recurrent tumors while still retaining significant associations (Pearson correlation, R= 0.51, p= 0.04) (**Figure 5B**). This reversal in correlation pattern was not observed for *CDLN4* and *CLDN7* for which there was no significant correlation with *CD44* expression in the primary OCs. However, the matched recurrent tumor samples did exhibit a strong positive correlation between *CD44* and both *CLDN4* and *CLDN7* (Pearson correlation, R= 0.58, p= 0.002 and R= 0.61, p= 0.01, respectively) (**Figure 5B**). Notably, there was no significant relationship between *CLDN1* and *CD133* in the primary and recurrent samples (Pearson correlation, R= −0.18, p= 0.50 and R= 0.04, p= 0.88, respectively; data not shown). These results suggest that the switch in the direction of correlation between *CLDN1* and *CD44* in matched primary—recurrent tumors plays an important biological role in tumor recurrence, as does the strengthened and significant positive correlation between *CD44* and *CLDN4* and *CLDN7* that is only apparent in recurrent disease.

**Figure 6:**
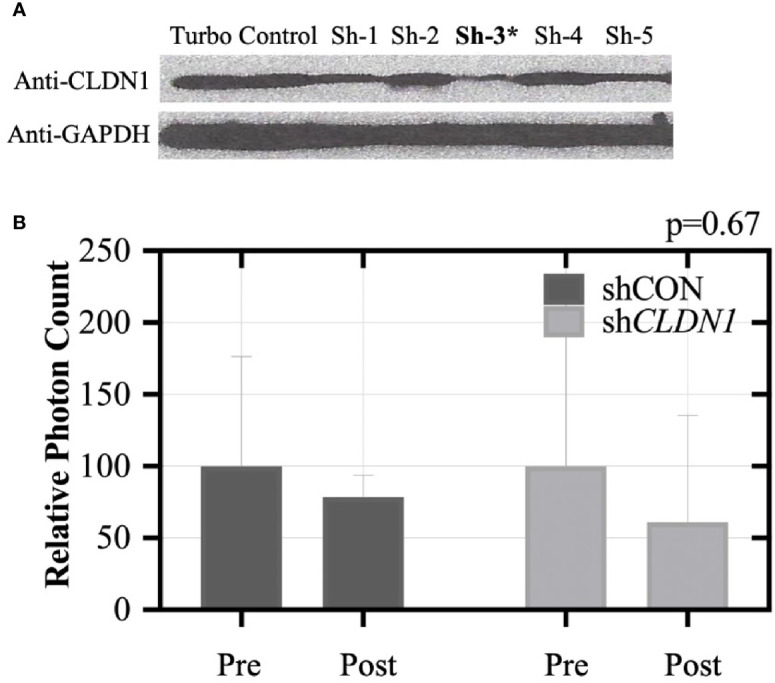
Claudin-1 (*CLDN1*) knockdown inhibits xenograft tumor formation in nude mice. **(A)** SKOV3 cells were stably transduced with shRNA for *CLDN1* or an off-target shRNA control using lentivirus infection. *CLDN1* protein expression was tested by Western blotting. Clone #3 was selected for use in the xenograft experiment, due to superior knockdown efficiency. Ten mice each were included in the control and *CLDN1* knockdown arms. SKOV3 cells (5×10^4^ per mouse) were injected intraperitoneally. **(B)** Tumor signal was monitored using the IVIS Kinetic Living Image System indicated as total photon flux. Bar graph shows relative signal strength for shCON and sh*CLDN1* tumors pre and post-treatment with carboplatin. After three treatments, 21.6% tumor size reduction in the control group and 39.1% tumor size reduction in the sh*CLDN*1 group have been observed (Mann-Whitney test, p = 0.67).

***Reduced CLDN1 Expression Inhibits Tumor Growth in Mice.*** To test the importance of *CLDN*1 to tumors *in vivo*, we injected female athymic nude mice with SKOV3 cells that had been transduced with a control shRNA or *CLDN1*-specific shRNA along with a GFP construct (GFP^+^/sh*CLDN*1 or GFP^+^/shControl (shCON), respectively). *CLDN1* knockdown was confirmed using Western Blot (**Figure 6A**). Six mice in each test group developed tumors of at least 1 × 10^7^ total photon flux *via* live imaging and received chemotherapeutic treatment with carboplatin. After three treatments, all six mice in the shCON group still had detectable tumors, but only two mice in the sh*CLDN*1 group had detectable tumors upon. The mice were dissected following the experiment to confirm tumor formation. A repeat test with the same experimental parameters showed greater average tumor size reduction in the sh*CLDN1* group versus the shCON group following treatment (39.1% versus 21.6%, respectively; **Figure 6B**), but the result was not statistically significant (Mann-Whitney test, p= 0.67). Taken together, these results support that repression of *CLDN1* expression in OC cells increases chemosensitivity *in vivo.*

## Discussion

The aim of this study was to evaluate the functional significance of *CLDN1* expression by using data from matched primary-recurrent OC tumor samples along with *in vitro* and *in vivo* studies. OC has a high mortality rate largely due to the fact that 75% of patients are diagnosed with advanced stage disease that has metastasized throughout the peritoneal cavity (44), and therefore an understanding of the mechanisms of metastasis is critical. OC metastasis occurs mainly through direct seeding of adjacent organs and tissues with cancer cells that have dissociated from the primary site (45). The role of tight junctions in cancer metastasis and recurrence is largely attributable to their function in cell-cell adhesion. *CLDNs* have emerged as a primary mediator in these cell-cell interactions and thus, cancer metastasis and recurrence (46). *CLDN1* has been studied in cancer progression (47) and an association between *CLDN1* expression and poor prognosis or survival was reported in colon (48) and breast cancers (49). In the short- and long-term survivor (GSE51820) dataset (21) from patients with post-chemotherapy ovarian carcinoma effusions, higher *CLDN1* expression was correlated with shorter overall survival (50). However, the molecular mechanisms by which *CLDN1* affects tumorigenesis and tumor progression in OC remain largely unstudied. Thus, the question of whether *CLDN1* functions as a tumor promoter or tumor suppressor has not been established in cancers, including OC.

An inverse association between DNA hypermethylation and gene expression of *CLDNs* has been identified in gastric cancer (16) and breast cancer cells (17). Di Cello et al. reported a negative correlation between *CLDN1* expression and promoter methylation in estrogen receptor-positive breast cancer (51). These data suggest that epigenetic regulation is involved in *CLDN* gene expression. The lack of mutations and wide range of gene expression levels in OCs from the TCGA data analysis further support our hypothesis that *CLDN1* may be regulated by epigenetic mechanisms. Indeed, we found that the expression of *CLDN1* was inversely correlated with DNA methylation in OC cells using both RT-PCR and microarray analysis. The results indicate that increased DNA methylation is associated with decreased *CLDN1* expression and this association seems to be important for OC recurrence (**Table 2**). Furthermore, this relationship was also observed in tumors from patients with short-term survival but not in tumors from patients with long-term survival. Additionally, patient survival was not associated with *CLDN1* expression in the matched primary-recurrent data. This suggests that methylation may regulate *CLDN1* expression in tumors from specific patients, and that it may play an important role in OC progression and prognosis. These data also show that epigenetic regulation of *CLDN1* expression is more pronounced in aggressive OCs, with stronger methylation-expression relationships potentially predictive of poorer patient prognosis.

Given that *CLDN1* is located in an intron of *CLDN16*, we hypothesized that there may be a similar relationship with *CLDN16* gene expression and methylation in aggressive OCs. The TCGA dataset showed a significant inverse correlation between methylation and expression of *CLDN16*, but this relationship was not detected in our primary-recurrent dataset. Interestingly, the sole intragenic *CLDN4* CpG site on this assay showed a significant inverse correlation in the recurrent tumors, yet DNMT inhibition of *CLDN4* showed no significant change in expression levels. Meanwhile, the *CLDN1* CpGs analyzed here showed significant inverse methylation-expression relationships in recurrent tumors, and DNMT treatment showed increased *CLDN1* expression levels. This may indicate that CpG islands are more resistant to the effects of DNMT inhibitors than CpG sites located in shore and shelf regions.

Our functional assessment of *CLDN1* in cancer cell proliferation, migration, spheroid formation, and wound healing also suggests that elevated *CLDN1* expression is associated with a more aggressive phenotype. Spheroid formation in ascites fluid is a fundamental feature of CICs (52) that is believed to allow cancer cells to embed themselves in surrounding tissues of the peritoneal cavity, which may seed metastases and may serve as precursors of recurrent disease. YM201636 and *CLDN1*-specific siRNAs both impede spheroid formation and suggest a potential role for targeting spheroids in order to limit OC spread and recurrence (**Figures 2C** and **3D** **)**. Disrupting the ability of OC cells to aggregate may prevent spheroids from embedding into other tissues in the peritoneal cavity and could also enhance sensitivity to chemotherapeutic agents.

We showed that *CLDN1* transcription is positively correlated with the expression of CIC marker CD44 in primary OC, but negatively correlated in recurrent OC (**Figure 5B**). Notably, the positive correlation between CD44 and *CLDN*1 expression in recurrent OC (**Figure 5B**) was supported by the decrease in CD44 and CD133 expression in *CLDN1* knockdown cells **Figure 5A**). This suggests a functional relationship between tight junction proteins and CICs, which lead to more aggressive and recurrent OCs.

Epithelial mesenchymal transition (EMT) and the reverse process, mesenchymal epithelial transition (MET), have been identified as important steps in cancer metastasis. Suh et al., showed that *CLDN1* is overexpressed in human hepatocellular carcinoma cells and is capable of promoting the EMT process, suggesting a close relationship between *CLDN1* and EMT (11). However, we did not find strong associations between *CLDN*1 and EMT factors using Western Blotting and immunohistochemistry. Similarly, the EMT RT^2^ Profiler PCR Arrays did not show significant changes for any primary EMT-related factors in *CLDN*1 knockdown cells. This indicates that the role of *CLDN1* in tumor metastasis and in the enhanced presence of CIC markers CD44 and CD133 is not due to EMT pathway induction.

Increased sensitivity to common chemotherapeutic agents for serous OC was observed in *CLDN1* knockdown OC cells, suggesting an association between *CLDN1* expression and chemosensitivity. Our *in vivo* study demonstrated that *CLDN1* expression is associated with greater tumor burden and resistance to treatment, though this result was not statistically significant. This was consistent with our *in vitro* model which showed greater chemoresistance in cells that expressed *CLDN1*. These preclinical results indicate *CLDN1* suppression may be a viable strategy for reducing the onset of aggressive OC phenotypes and recurrent disease. Our use of YM201636, which blocks recycling of tight junction proteins, demonstrates its efficacy in limiting CLDN1 function and provides a potential treatment strategy for more advanced OC.

Our findings may provide new targets for OC treatment, specifically with the aim of extending survival by preventing recurrent OC. For example, new technology like that provided by using a deactivated dCas9 (53) that can specifically methylate the *CLDN1* promoter sequence and inhibit transcriptional activity of *CLDN1* presents an exciting opportunity. Thus, *CLDN1* targeting may be an effective treatment option for recurrent OC, particularly in patients with high basal *CLDN1* expression levels. The idea to reduce expression of *CLDN1* should also be considered in the context of the use of general DNA hypomethylating agents that are being used to reactivate tumor suppressor and pro-apoptotic genes and potentiate response to other cytotoxic chemotherapeutic agents, including in solid tumors. Such treatment could also inadvertently reactivate expression of genes like *CLDN1* for which increasing expression may be detrimental. In this regard, the development of highly targeted, individualized approaches to reactivate as well as repress particular genes based on their status will be optimal but must await further technological advances that allow for such a strategy to be clinically implemented.

This study’s strengths include the use of paired primary-recurrent tumors and ‘omics technologies to identify methylation/expression relationships that are divergent between these two phases of the disease. This study is also strengthened by the use of both *in vitro* and *in vivo* studies to validate the role of *CLDN1* in OC. This study’s limitations include a relatively small sample size and the retrospective nature of the analysis.

Taken together, our results support that *CLDN1* plays a critical role in the development of recurrent OC and resistance to current chemotherapeutic regimens that are standard of care. Developing strategies to target *CLDN*1 may lead to enhanced chemosensitivity and hinder tumor metastasis and OC recurrence.

## Data Availability Statement

The raw data supporting the conclusions of this article is available, without undue reservation from the Duke Digital Data Repository.

## Ethics Statement

The studies involving human participants were reviewed and approved by Duke University Institutional Review Board. The patients/participants provided their written informed consent to participate in this study. The animal study was reviewed and approved by Duke Institutional Animal Care and Use Committee.

## Author Contributions

ZV, AB, SM, and ZH performed project development and experimental design. ZV, GS, CG, M-HB, SS, IR, RW, DY, and ZH performed data collection. ZV and ZH performed data analysis and SM original manuscript drafting. AB, SM, and GS provided funding support. ZV, IR, AB, SM, and ZH performed manuscript editing and revision. All authors contributed to the article and approved the submitted version.

## Funding

This study was supported by the Foundation for Women’s Cancer (GS and SM) and the Gail Parkins Ovarian Cancer Awareness Fund.

## Conflict of Interest

The authors declare that the research was conducted in the absence of any commercial or financial relationships that could be construed as a potential conflict of interest.

## Supplementary Material

Supplementary Figure 1
*CLDN1* knockdown efficiency in seven OC cell lines 72 hours post-transfection with beta-2-microglobulin as an internal control. HEYA8 had the greatest knockdown efficiency at approximately 88%.

Supplementary Figure 2 Two siRNA variants, si*CLDN1-6* and si*CLDN1-8* were transfected into IGROV1 and CAOV2 cells to identify which variant produced a more effective *CLDN1* knockdown compared to siCON (p = two-sample t-test; *p < 0.05, **p < 0.01).

Supplementary Figure 3
**(A)** *CLDN1* methylation-expression relationships in 16 matched primary-recurrent ovarian cancer tumor pairs at four CpG sites. The positions of the CpG probes on the Illumina Infinium HumanMethylation450 BeadChip *CLDN1* locus are shown in the schematic in **Figure 1**. **(B)** *CLDN1* methylation-expression relationships in 21 long-term survival (>7 years) and 26 short-term survival (<3 years) ovarian cancer patients. X-axis, Illumina Infinium HumanMethylation450 BeadChip methylation values; 0, unmethylated; 1, fully methylated. Y-axis, RMA normalized *CLDN1* expression values. R, Pearson correlation coefficient; p, correlation p-value.

Supplementary Table 1 Microarray data showing CLDN1 expression for 26 cell lines treated with decitabine versus mock control.

Supplementary Table 2 EMT RT^2^ Profiler PCR Array results for 84 EMT-associated genes following *CLDN1* knockdown.
