# Supplementary figures and images for: Epigenetic Regulation of Claudin-1 in the Development of Ovarian Cancer Recurrence and Drug Resistance
Source: Front Oncol. 2021 Mar 22;11:620873. doi: 10.3389/fonc.2021.620873 (PMC8019902; doi:10.3389/fonc.2021.620873)

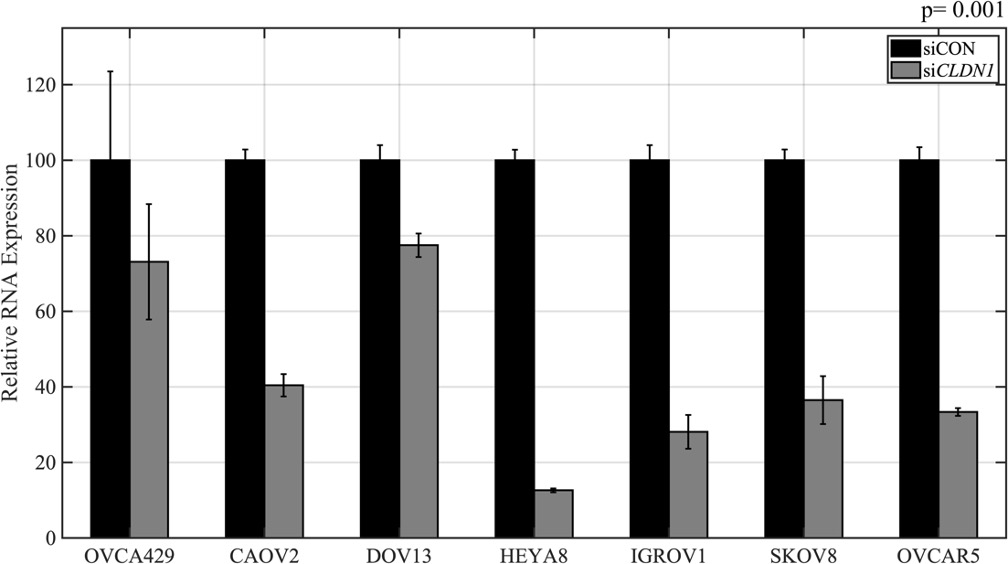

Supplement: Supplementary Figure 1 — CLDN1 knockdown efficiency in seven OC cell lines 72 hours post-transfection with beta-2-microglobulin as an internal control. HEYA8 had the greatest knockdown efficiency at approximately 88%. [file Image_1.jpeg]

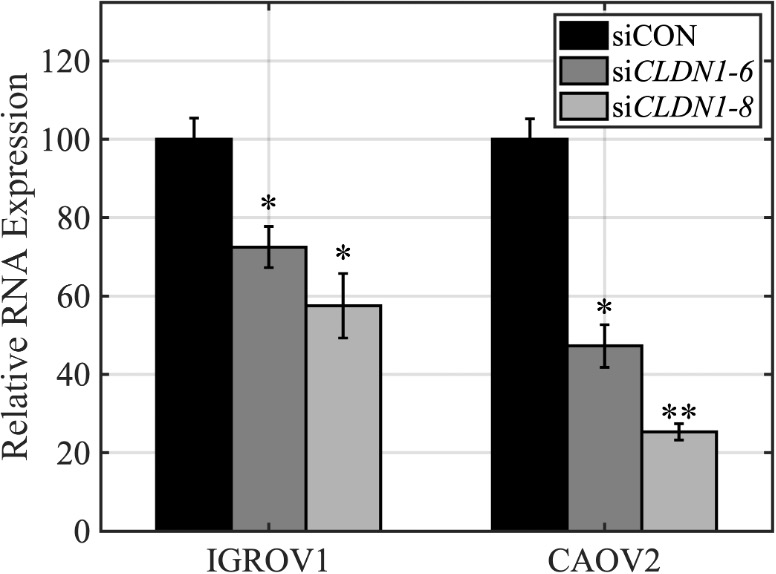

Supplement: Supplementary Figure 2 — Two siRNA variants, siCLDN1-6 and siCLDN1-8 were transfected into IGROV1 and CAOV2 cells to identify which variant produced a more effective CLDN1 knockdown compared to siCON (p = two-sample t-test; *p < 0.05, **p < 0.01). [file Image_2.jpeg]

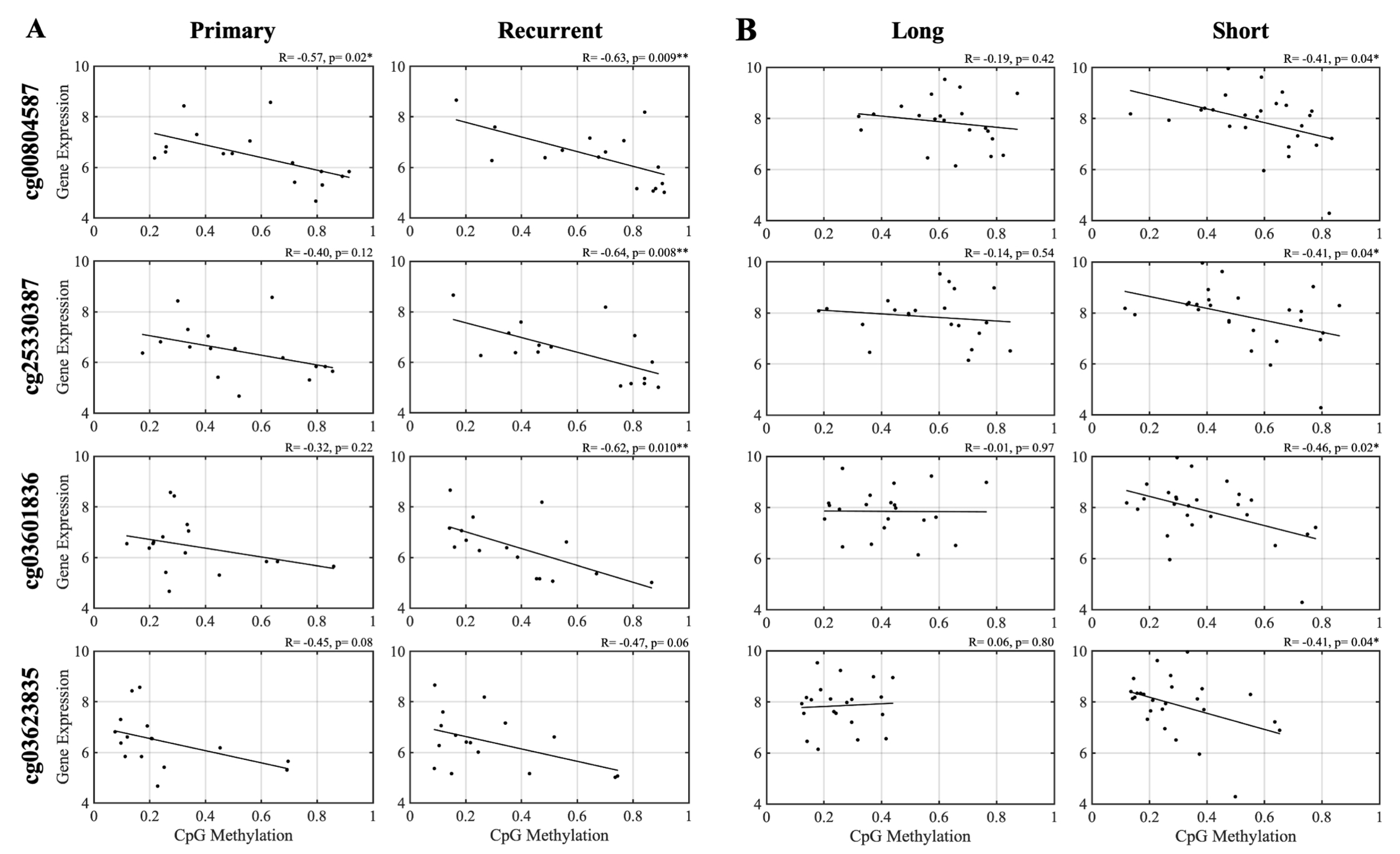

Supplement: Supplementary Figure 3 — (A) CLDN1 methylation-expression relationships in 16 matched primary-recurrent ovarian cancer tumor pairs at four CpG sites. The positions of the CpG probes on the Illumina Infinium HumanMethylation450 BeadChip CLDN1 locus are shown in the schematic in Figure 1 . (B) CLDN1 methylation-expression relationships in 21 long-term survival (>7 years) and 26 short-term survival (<3 years) ovarian cancer patients. X-axis, Illumina Infinium HumanMethylation450 BeadChip methylation values; 0, unmethylated; 1, fully methylated. Y-axis, RMA normalized CLDN1 expression values. R, Pearson correlation coefficient; p, correlation p-value. [file Image_3.jpeg]
