# Supplementary material for: Epigenetic Regulation of Claudin-1 in the Development of Ovarian Cancer Recurrence and Drug Resistance
Source: Front Oncol. 2021 Mar 22;11:620873. doi: 10.3389/fonc.2021.620873 (PMC8019902; doi:10.3389/fonc.2021.620873)
Supplement: Supplementary Table 1 — Microarray data showing CLDN1 expression for 26 cell lines treated with decitabine versus mock control. [file Table_1.docx]

| **SUPPLEMENTARY TABLE 1. Microarray data showing CLDN1 expression for 26 cell lines treated with decitabine versus mock control.** | | | | | |
| --- | --- | --- | --- | --- | --- |
| **Cell Line** | **Mock** | **Decitabine** | **Cell Line** | **Mock** | **Decitabine** |
| TOV21 | 11.2 | 6.7 | Tyknu | 223.4 | 657.3 |
| A2780 | 15.3 | 3.9 | HEYA8 | 263.7 | 1189 |
| CAOV3 | 15.8 | 4 | SKOV3 | 282 | 466 |
| Tyknu-cisR | 40.3 | 276.9 | DOV13 | 296 | 395 |
| OVARY1847 | 44.3 | 50.9 | OVCA429 | 343.5 | 786 |
| A2780-cisR | 99 | 110.4 | OV90 | 426.4 | 288.9 |
| M41-cisR | 130.5 | 557.6 | M41 | 432.5 | 1508.6 |
| OVCA433 | 138.1 | 374.4 | HEY | 626.3 | 1252 |
| PEO4 | 151.9 | 278.9 | OVCAR5 | 721.3 | 1159.7 |
| CAOV2 | 155.5 | 244.3 | PEO1 | 724.2 | 2727.3 |
| OVCAR2 | 179.8 | 332 | OVCA420 | 875.2 | 1853.6 |
| OVCA432 | 220 | 1286.3 | HEYC2 | 888.1 | 2759.7 |
| OVCAR3 | 223.1 | 668.4 | SKOV8 | 3957.2 | 4245.4 |
