# Supplementary material for: Epigenetic Regulation of Claudin-1 in the Development of Ovarian Cancer Recurrence and Drug Resistance
Source: Front Oncol. 2021 Mar 22;11:620873. doi: 10.3389/fonc.2021.620873 (PMC8019902; doi:10.3389/fonc.2021.620873)
Supplement: Supplementary Table 2 — EMT RT2 Profiler PCR Array results for 84 EMT-associated genes following CLDN1 knockdown. [file Table_2.docx]

| SUPPLEMENTARY TABLE 2. EMT RT^2^ Profiler PCR Array results for 84 EMT-associated genes following *CLDN1* knockdown. | | | |
| --- | --- | --- | --- |
| **Refseq** | **Symbol** | **Description** | **Fold Change** |
| NM_024060 | AHNAK | AHNAK nucleoprotein | 1.4 |
| NM_006129 | BMP1 | Bone morphogenetic protein 1 | 1.6 |
| NM_004342 | CALD1 | Caldesmon 1 | 0.7 |
| NM_018584 | **CAMK2N1** | **Calcium/calmodulin-dependent protein kinase II inhibitor 1** | **2.3** |
| NM_001792 | CDH2 | Cadherin 2, type 1, N-cadherin (neuronal) | 1.2 |
| NM_000089 | COL1A2 | Collagen, type I, alpha 2 | 0.6 |
| NM_000090 | COL3A1 | Collagen, type III, alpha 1 | 1.0 |
| NM_000393 | COL5A2 | Collagen, type V, alpha 2 | 1.7 |
| NM_002026 | FN1 | Fibronectin 1 | 1.7 |
| NM_005251 | FOXC2 | Forkhead box C2 (MFH-1, mesenchyme forkhead 1) | 1.8 |
| NM_004126 | GNG11 | Guanine nucleotide binding protein (G protein), gamma 11 | 1.8 |
| NM_173849 | **GSC** | **Goosecoid homeobox** | **2.3** |
| NM_001552 | IGFBP4 | Insulin-like growth factor binding protein 4 | 1.6 |
| NM_002205 | ITGA5 | Integrin, alpha 5 (fibronectin receptor, alpha polypeptide) | 1.1 |
| NM_002210 | ITGAV | Integrin, alpha V (vitronectin receptor, alpha polypeptide, antigen CD51) | 1.6 |
| NM_004530 | MMP2 | Matrix metallopeptidase 2 (gelatinase A, 72kDa gelatinase, 72kDa type IV collagenase) | 2.0 |
| NM_002422 | MMP3 | Matrix metallopeptidase 3 (stromelysin 1, progelatinase) | 1.0 |
| NM_004994 | MMP9 | Matrix metallopeptidase 9 (gelatinase B, 92kDa gelatinase, 92kDa type IV collagenase) | 1.2 |
| NM_002444 | MSN | Moesin | 1.1 |
| NM_000602 | SERPINE1 | Serpin peptidase inhibitor, clade E (nexin, plasminogen activator inhibitor type 1), member 1 | 0.5 |
| NM_005985 | SNAI1 | Snail homolog 1 (Drosophila) | 1.9 |
| NM_003068 | SNAI2 | Snail homolog 2 (Drosophila) | 1.4 |
| NM_178310 | SNAI3 | Snail homolog 3 (Drosophila) | 1.6 |
| NM_006941 | SOX10 | SRY (sex determining region Y)-box 10 | 1.0 |
| NM_003118 | **SPARC** | **Secreted protein, acidic, cysteine-rich (osteonectin)** | **2.1** |
| NM_012449 | STEAP1 | Six transmembrane epithelial antigen of the prostate 1 | 1.0 |
| NM_003199 | TCF4 | Transcription factor 4 | 1.0 |
| NM_003254 | TIMP1 | TIMP metallopeptidase inhibitor 1 | 1.6 |
| NM_003692 | TMEFF1 | Transmembrane protein with EGF-like and two follistatin-like domains 1 | 1.0 |
| NM_178031 | TMEM132A | Transmembrane protein 132A | 1.7 |
| NM_000474 | TWIST1 | Twist homolog 1 (Drosophila) | 1.5 |
| NM_004385 | VCAN | Versican | 1.3 |
| NM_003380 | VIM | Vimentin | 1.2 |
| NM_033305 | VPS13A | Vacuolar protein sorting 13 homolog A (S. cerevisiae) | 0.9 |
| NM_003392 | WNT5A | Wingless-type MMTV integration site family, member 5A | 1.9 |
| NM_032642 | WNT5B | Wingless-type MMTV integration site family, member 5B | 1.3 |
| **Down-Regulated Genes During EMT** | | | |
| **Refseq** | **Symbol** | **Description** | **Fold Change** |
| NM_001233 | CAV2 | Caveolin 2 | 1.7 |
| NM_004360 | CDH1 | Cadherin 1, type 1, E-cadherin (epithelial) | 1.0 |
| NM_004415 | DSP | Desmoplakin | 1.9 |
| NM_005130 | **FGFBP1** | **Fibroblast growth factor binding protein 1** | **3.0** |
| NM_000577 | IL1RN | Interleukin 1 receptor antagonist | 1.0 |
| NM_002276 | KRT19 | Keratin 19 | 1.1 |
| NM_002447 | MST1R | Macrophage stimulating 1 receptor (c-met-related tyrosine kinase) | 1.0 |
| NM_015901 | NUDT13 | Nudix (nucleoside diphosphate linked moiety X)-type motif 13 | 0.9 |
| NM_002538 | OCLN | Occludin | 1.4 |
| NM_015704 | DESI1 | PPPDE peptidase domain containing 2 | 1.1 |
| NM_002923 | RGS2 | Regulator of G-protein signaling 2, 24kDa | 1.0 |
| NM_000582 | **SPP1** | **Secreted phosphoprotein 1** | **0.3** |
| NM_006528 | TFPI2 | Tissue factor pathway inhibitor 2 | 1.9 |
| NM_014399 | TSPAN13 | Tetraspanin 13 | 1.3 |
| *Bolded gene names and fold change indicate greater than 2-fold increase or half-fold decrease. | | | |
